# High Prevalence of Plasmid‐Mediated Quinolone Resistance in *Salmonella enterica* Serovars Isolated From Surface Water

**DOI:** 10.1111/1462-2920.70140

**Published:** 2025-07-07

**Authors:** Daniel F. M. Monte, Alan Douglas de Lima Rocha, Mateus Lacerda Pereira Lemos, Laiorayne Araújo de Lima, Julia Memrava Cabrera, Nádyra Jerônimo da Silva, Xinyang Huang, Zhao Chen, Eric W. Brown, Marc W. Allard, Rebecca L. Bell, Magaly Toro, Jianghong Meng, Celso José Bruno de Oliveira

**Affiliations:** ^1^ Department of Population Health and Pathobiology North Carolina State University, College of Veterinary Medicine Raleigh North Carolina USA; ^2^ Departmento de Zootecnia Centro de Ciências Agrárias, Universidade Federal da Paraíba (CCA/UFPB) Areia Paraíba Brazil; ^3^ Sao Paulo State University (Unesp) School of Agricultural and Veterinarian Sciences Jaboticabal São Paulo Brazil; ^4^ US Food and Drug Administration, Human Foods Program Office of Laboratory Operations and Applied Science College Park Maryland USA; ^5^ Joint Institute for Food Safety and Applied Nutrition (JIFSAN), University of Maryland, College Park College Park Maryland USA

**Keywords:** antimicrobial‐resistance, environmental water, genomic analysis

## Abstract

Considering the increasing reports of *Salmonella enterica* strains resistant to quinolones, antimicrobials frequently employed as therapeutic agents globally, our goal was to investigate the occurrence of plasmid‐mediated quinolone resistance (PMQR) determinants in *S. enterica* recovered from natural surface waters in Paraíba state, Brazil. Water samples (*n* = 230) were collected monthly in triplicate using modified Moore swabs from 29 sampling sites belonging to 10 large dams. After conventional microbial isolation, representative isolates (*n* = 938) were submitted to whole genome sequencing, assembly and annotation. Antimicrobial resistance genes (ARGs) were identified, and core genome multilocus sequence typing (cgMLST) was used to infer phylogenetic relationships. Among recovered *S. enterica* , 130 (13.9%) isolates harboured PMQR determinants; 124 (95.4%) harboured qnrB19, while 6 (4.6%) harboured qnrS1. Multiple other ARGs associated with resistance to aminoglycosides, β‐lactams, sulphonamides, tetracyclines and fosfomycin were identified. The diversity of ARGs and plasmids suggests a highly complex resistance landscape. Phylogenetic analysis revealed clustering by serovar and sequence type but not by resistance profile or geographic origin. The absence of association between phylogeny and ARGs highlights the potential role of horizontal gene transfer in disseminating resistance genes in water. Our findings reinforce the importance of antimicrobial resistance surveillance in surface waters.

## Graphical abstract

Our study reports a high occurrence of PMQR (plasmid‐mediated quinolone resistance) *Salmonella* in surface water, posing a significant public health risk as these resistant strains can contaminate foods through irrigation potentially leading to outbreaks of severe foodborne illness.

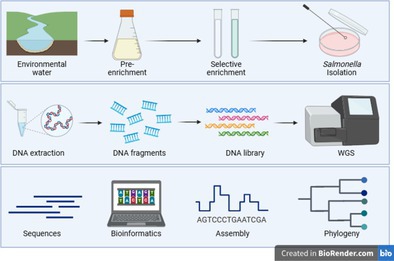

## 1 Introduction

Multidrug‐resistant (MDR) *Salmonella enterica* ( *S. enterica* ) strains pose a significant burden to global public health, as they are associated with foodborne illness with compromised therapeutic success, leading to elevated rates of clinical complications and mortality. Notably concerning is the increasing resistance to quinolones, antimicrobials frequently employed as primary or secondary therapeutic agents worldwide.

Quinolone resistance in *S. enterica* can arise from chromosomal point mutations in topoisomerase‐encoding genes or through horizontally transferable plasmid‐mediated quinolone resistance (PMQR) genetic elements, including the pentapeptide repeat proteins (*qnr* variants), the aminoglycoside acetyltransferase variant *aac(6′)‐Ib‐cr* and the efflux pump‐encoding *qepA*. These PMQR genetic elements facilitate the intra‐ and interspecies dissemination of resistance across bacterial populations and environments (Nordmann and Poirel 2005; Jacoby et al. 2014; Moreno‐Switt et al. 2019; Aworh et al. 2024), possibly explaining the increasing number of quinolone‐resistant *S. enterica* serovars (EFSA 2025).

Interestingly, studies indicate that the genes responsible for PMQR, particularly those of the *qnr* family, likely originated from microorganisms commonly found in water (Strahilevitz et al. 2009). Variants such as *qnr*A, *qnr*S and *qnr*D have chromosomal homologues in bacteria naturally found in aquatic environments, including *Shewanella algae* , *Vibrio splendidus* and *Aeromonas* spp. (Poirel et al. 2012). These non‐pathogenic microorganisms are considered natural reservoirs of these genes, which were likely transferred horizontally to enteric bacteria via mobile genetic elements such as plasmids, facilitating the spread of resistance in both clinical and environmental settings (Cattoir et al. 2008). Importantly, this can be associated with the increasing reports of PMQR‐mediated antimicrobial resistance in *S. enterica* recovered from natural water environments (Chen et al. 2024; Toyting et al. 2024).

The dissemination of PMQR determinants is particularly concerning in resource‐limited settings characterised by inadequate antimicrobial stewardship protocols and insufficient wastewater management infrastructure. Data from the 2022 Census indicate that 24.3% of the Brazilian population continues to experience inadequate access to sanitation infrastructure or effluent treatment facilities (IBGE 2023), leading to untreated waste discharge directly into water bodies and consequent environmental microbial contamination. This situation is particularly pronounced in semi‐arid geographical zones dependent exclusively on surface water impoundments for both human consumption and agro‐industrial operations, such as the state of Paraíba situated in Brazil's semi‐arid northeastern region. Such hydrological resource dependence intensifies anthropogenic pressure on watershed ecosystems, predominantly attributable to intensive agricultural production systems. Recent epidemiological surveillance conducted by our research consortium has documented a high *S. enterica* contamination rate (70.9%) in surface water from hydrographic basins in this region (Rocha et al. 2025).

This study aimed to investigate the presence and diversity of PMQR genes in *S. enterica* isolates from surface water ecosystems in Paraíba, Brazil. By comprehensive molecular characterisation of the resistance profiles and genetic context of these isolates, our research aimed at better understanding the regional epidemiological dynamics of antimicrobial resistance and contributing to global antimicrobial resistance surveillance initiatives.

## 2 Materials and Methods

### 2.1 Study Design and Sampling Sites Characterisation

**FIGURE 1:**
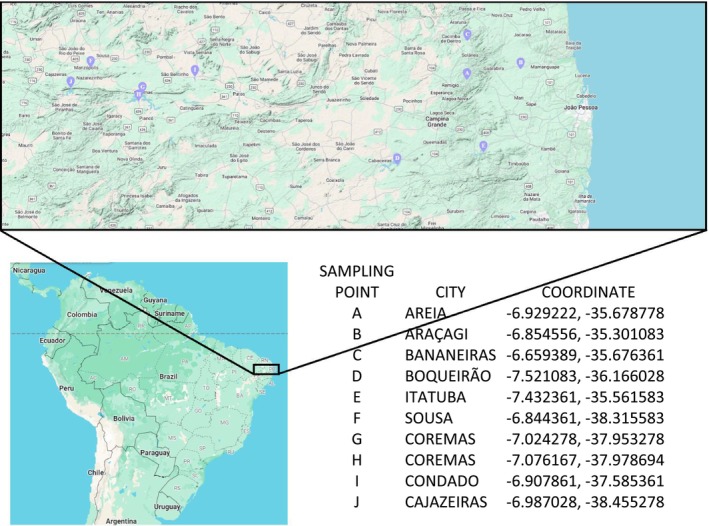
Geographical locations of the ten large dams (A to J) associated with the three largest hydrographic basins in Paraíba state, Brazil: the Piranhas, Paraíba and Mamanguape rivers.

We conducted a longitudinal study from January 2021 to May 2022, with samplings performed in January, February, March, April, May, June and July 2021, and January, February, March, April, and May 2022. Samples were taken from 29 sampling sites belonging to 10 large dams located within the three largest hydrographic basins in Paraíba state, Brazil: the Piranhas, Paraíba and Mamanguape rivers (Figure 1). Within each of the selected dams, three independent sampling sites were chosen near water margins where agricultural and livestock activities were prevalent. Samples were collected in triplicate from each site during each sampling event, resulting in 230 water samples (690 swabs).

Sixty‐three samples were collected from the Mamanguape river basin, which covers an area of 3522.69 km^2^. Its headwaters are located in the central region of the state and flow northeastward, ultimately discharging into the Atlantic Ocean (AESA 2017, 2022). Sampling sites in this basin included: two points in the Araçagi reservoir, located on the Mamanguape River, and one point on the Jacaré River, all within the municipality of Araçagi; three points in the Saulo Maia dam in the municipality of Areia; and two points in the Jandáia dam in the municipality of Bananeiras.

Forty‐eight samples were collected from the Paraíba river basin, which spans 20,071.83 km^2^. It originates in the south‐central part of the state and flows eastward, emptying into the Atlantic Ocean (AESA 2017, 2022). Although it is the second largest basin in the state, it holds significant economic importance, supplying water to 52% of the state's population (AESA 2017). Samples were collected from three points in the Epitácio Pessoa dam, the second largest in the state, with a capacity of 466 million m^3^. Two additional sampling points were established in the Acauã dam, the fourth largest in the state, with a capacity of 253 million m^3^, and one sampling point was located along the Paraíba river.

One hundred and nineteen samples were collected from the Piranhas river basin, the largest in the state, covering 26,047.49 km^2^. Its headwaters rise in the southwestern region of the state and flow northeast, discharging into the Atlantic Ocean in the northern part of the neighbouring state of Rio Grande do Norte (AESA 2017, 2022). In the municipality of Coremas, three sampling points were selected in the Coremas dam and two in the Mãe D'água dam. These interconnected reservoirs together form the largest water storage system in the state, with a combined capacity of 1.289 billion m^3^. Three additional sampling points were established in the São Gonçalo dam, located in the municipality of Sousa. Another three were selected in the Engenheiro Ávidos dam, the third largest in the state, with a capacity of 293 million m^3^, located in the municipality of Cajazeiras. In the municipality of Condado, three more sampling points were selected in the Engenheiro Arcoverde dam.

### 2.2 Samplings and Microbiological Procedures

Water samples were collected using modified Moore Swabs (MMS) as previously described (Sbodio et al. 2013). This sampling method has been chosen for its high efficiency in capturing *S. enterica* from large volumes of water with low bacterial loads, making it well suited for environmental surveillance in surface waters impacted by agricultural runoff (Bisha et al. 2014).

Each swab consisted of a 0.9 m^2^ folded cheesecloth rolled into an MMS cassette, which was sterilised by autoclaving before use. The MMS units were connected to a portable peristaltic pump, and 10 L of water samples were filtered at a rate of 500 mL/min for 20 min. After sampling, the swabs were transferred into sterile flasks containing 250 mL of modified buffered peptone water and kept at 4°C during transport to the laboratory.

*S. enterica* isolation was performed according to Andrews et al. (2024) with minor modifications. Swabs were pre‐enriched in modified buffered peptone water at 37°C ± 1°C for 24 h and then inoculated into Rappaport‐Vassiliadis (RV) and tetrathionate (TT) broths. After incubation at 42.5°C ± 1°C for 18 h, samples were streaked onto XLT‐4 agar and subjected to broth polymerase chain reaction (PCR) targeting the *inv*A gene. PCR‐positive samples were further streaked onto Hektoen enteric agar and bismuth sulphite agar. Up to three presumptive *S. enterica* colonies were subjected to biochemical tests using triple sugar iron (TSI) and lysine iron agar (LIA) slants. The isolates were confirmed by PCR and stored in brain heart infusion (BHI) broth with glycerol at −80°C.

DNA for PCR analysis of both enrichment broths and bacterial isolates was extracted using the boiling‐centrifugation protocol described by Freschi et al. (2005). The *invA* gene was targeted using forward (5′‐GTG AAA TTA TCG CCA CGT TCG GGC AA‐3′) and reverse (5′‐TCA TCG CAC CGT CAA AGG AAC C‐3′) primers, with reaction composition and thermal cycling parameters following the methodology established by Rocha et al. (2025).

### 2.3 Selection of *Salmonella* Strains for Whole Genome Sequencing (WGS)

Up to four isolates originating from a single MMS, preferably from different media, were subjected to whole‐genome sequencing (WGS). These isolates were derived from water samples collected across multiple sampling periods, ensuring a comprehensive representation of the aquatic microbial community over the study period (Table 1).

**TABLE 1:** Genomic features of plasmid‐mediated quinolone‐resistant *Salmonella enterica* strains.

Accession number	Serovar by WGS	Source	Resistance genes	Sequence type	Location	Plasmid
GCA_029744145.1	Anatum	Dam water	*mdsA, mdsB, qnrB19*	64	7°25′56.5″ S 35°33′41.7″ W	Col156, Col(pHAD28)
GCA_029744265.1	Anatum	Dam water	*mdsA, mdsB, qnrB19*	64	7°25′56.5″ S 35°33′41.7″ W	Col156, Col(pHAD28)
GCA_029746855.1	Agona	River	*fosA7.2, mdsA, mdsB, qnrB19*	13	6°51′47.1″ S 35°21′32.1″ W	Col(pHAD28)
GCA_029744445.1	Agona	River	*fosA7.2, mdsA, mdsB, qnrB19*	13	6°51′27.8″ S 35°22′02.5″ W	Col(pHAD28)
GCA_029746835.1	Agona	River	*fosA7.2, mdsA, mdsB, qnrB19*	13	6°51′47.1″ S 35°21′32.1″ W	Col(pHAD28)
GCA_029688465.1	Schwarzengrund	Dam water	*mdsA, mdsB, qnrB19*	96	7°01′34.9″ S 37°56′36.7″ W	Col(pHAD28)
GCA_029670245.1	Muenchen	Dam water	*mdsA, mdsB, qnrB19*	112	6°51′22.5″ S 38°21′05.5″ W	Col(pHAD28)
GCA_029670285.1	Muenchen	Dam water	*mdsA, mdsB, qnrB19*	112	6°51′22.5″ S 38°21′05.5″ W	Col(pHAD28)
GCA_029670345.1	Schwarzengrund	Dam water	*aph(3″)‐Ib, aph(6)‐Id, mdsA, mdsB, qnrB19, sul2, tet(A)*	96	6°50′45.1″ S 38°18′40.1″ W	Col(pHAD28)
GCA_029670405.1	Schwarzengrund	Dam water	*aph(3″)‐Ib, aph(6)‐Id, mdsA, mdsB, qnrB19, sul2, tet(A)*	96	6°50′45.1″ S 38°18′40.1″ W	Col(pHAD28)
GCA_029671225.1	Javiana	Dam water	*mdsA, mdsB, qnrB19*	1674	6°51′22.5″ S 38°21′05.5″ W	Col(pHAD28)
GCA_029671365.1	Schwarzengrund	Dam water	*aph(3″)‐Ib, aph(6)‐Id, mdsA, mdsB, qnrB19, sul2, tet(A)*	96	6°50′45.1″ S 38°18′40.1″ W	Col(pHAD28)
GCA_029670525.1	Schwarzengrund	Dam water	*aph(3″)‐Ib, aph(6)‐Id, mdsA, mdsB, qnrB19, sul2, tet(A)*	96	6°50′45.1″ S 38°18′40.1″ W	Col(pHAD28)
GCA_029670485.1	Schwarzengrund	Dam water	*mdsA, mdsB, qnrB19*	96	6°50′45.1″ S 38°18′40.1″ W	Col(pHAD28)
GCA_029671525.1	Schwarzengrund	Dam water	*aph(3″)‐Ib, aph(6)‐Id, mdsA, mdsB, qnrB19, sul2, tet(A)*	96	6°50′45.1″ S 38°18′40.1″ W	Col(pHAD28)
GCA_029670545.1	Schwarzengrund	Dam water	*aph(3″)‐Ib, aph(6)‐Id, mdsA, mdsB, qnrB19, sul2, tet(A)*	96	6°50′45.1″ S 38°18′40.1″ W	Col(pHAD28)
GCA_029670645.1	Schwarzengrund	Dam water	*aph(3″)‐Ib, aph(6)‐Id, mdsA, mdsB, qnrB19, sul2, tet(A)*	96	6°50′45.1″ S 38°18′40.1″ W	Col(pHAD28)
GCA_029672065.1	Muenchen	Dam water	*mdsA, mdsB, qnrB19*	112	6°51′22.5″ S 38°21′05.5″ W	Col(pHAD28)
GCA_029672045.1	Schwarzengrund	Dam water	*aph(3″)‐Ib, aph(6)‐Id, mdsA, mdsB, qnrB19, sul2, tet(A)*	96	6°50′45.1″ S 38°18′40.1″ W	Col(pHAD28)
GCA_029672185.1	Muenchen	Dam water	*mdsA, mdsB, qnrB19*	112	6°51′22.5″ S 38°21′05.5″ W	Col(pHAD28)
GCA_029672105.1	Javiana	Dam water	*mdsA, mdsB, qnrB19*	1674	6°51′22.5″ S 38°21′05.5″ W	Col(pHAD28)
GCA_029672305.1	Javiana	Dam water	*mdsA, mdsB, qnrB19*	1674	6°51′22.5″ S 38°21′05.5″ W	Col(pHAD28)
GCA_029672685.1	Javiana	Dam water	*mdsA, mdsB, qnrB19*	1674	6°39′35.5″ S 35°40′34.0″ W	Col(pHAD28)
GCA_029672785.1	Schwarzengrund	Dam water	*aph(3″)‐Ib, aph(6)‐Id, mdsA, mdsB, qnrB19, sul2, tet(A)*	96	6°50′45.1″ S 38°18′40.1″ W	Col(pHAD28)
GCA_029672745.1	Schwarzengrund	Dam water	*aph(3″)‐Ib, aph(6)‐Id, mdsA, mdsB, qnrB19, sul2, tet(A)*	96	6°50′45.1″ S 38°18′40.1″ W	Col(pHAD28)
GCA_029672725.1	Schwarzengrund	Dam water	*aph(3″)‐Ib, aph(6)‐Id, mdsA, mdsB, qnrB19, sul2, tet(A)*	96	6°50′45.1″ S 38°18′40.1″ W	Col(pHAD28)
GCA_031380575.1	Muenchen	Dam water	*mdsA, mdsB, qnrB19*	112	7°25′57.0″ S 35°33′39.2″ W	Col(pHAD28)
GCA_031380605.1	Muenchen	Dam water	*mdsA, mdsB, qnrB19*	112	7°25′57.0″ S 35°33′39.2″ W	Col(pHAD28)
GCA_031380645.1	Javiana	Dam water	*mdsA, mdsB, qnrB19*	1674	7°25′57.0″ S 35°33′39.2″ W	Col(pHAD28)
GCA_031381475.1	Anatum	River	*mdsA, mdsB, qnrB19*	64	6°51′27.8″ S 35°22′02.5″ W	Col(pHAD28)
GCA_029755695.1	Minnesota	River	*aph(3′)‐Ia, bla*_*CMY‐2*_, *mdsA, mdsB, qnrB19, sul2*, *tet(A)*	548	6°51′27.8″ S 35°22′02.5″ W	IncC, Col(pHAD28)
GCA_029754055.1	Corvallis	River	*mdsA, mdsB, qnrB19*	1541	6°51′47.1″ S 35°21′32.1″ W	Col(pHAD28)
GCA_029754395.1	Javiana	River	*mdsA, mdsB, qnrB19*	1674	6°51′47.1″ S 35°21′32.1″ W	Col(pHAD28)
GCA_029754975.1	Corvallis	River	*mdsA, mdsB, qnrB19*	1541	6°51′47.1″ S 35°21′32.1″ W	Col(pHAD28)
GCA_029756035.1	Javiana	River	*mdsA, mdsB, qnrB19*	1674	6°51′47.1″ S 35°21′32.1″ W	Col(pHAD28)
GCA_029754595.1	Agona	River	*fosA7.2, mdsA, mdsB, qnrB19*	13	6°51′47.1″ S 35°21′32.1″ W	Col(pHAD28)
GCA_029753415.1	Muenchen	Dam water	*mdsA, mdsB, qnrB19*	112	6°50′45.1″ S 38°18′40.1″ W	Col(pHAD28)
GCA_029756055.1	Schwarzengrund	Dam water	*mdsA, mdsB, qnrB19*	96	6°50′45.1″ S 38°18′40.1″ W	ColpVC, Col(pHAD28)
GCA_029752055.1	Muenchen	Dam water	*mdsA, mdsB, qnrB19*	112	6°50′45.1″ S 38°18′40.1″ W	Col(pHAD28)
GCA_029751425.1	Corvallis	River	*mdsA, mdsB, qnrB19*	1541	6°51′47.1″ S 35°21′32.1″ W	Col(pHAD28)
GCA_029751615.1	Muenchen	Dam water	*mdsA, mdsB, qnrB19*	112	6°50′39.7″ S 38°18′56.1″ W	Col(pHAD28)
GCA_029748215.1	Cerro	River	*mdsA, mdsB, qnrB19*	367	6°51′47.1″ S 35°21′32.1″ W	Col(pHAD28)
GCA_029748175.1	Cerro	River	*mdsA, mdsB, qnrB19*	367	6°51′47.1″ S 35°21′32.1″ W	Col(pHAD28)
GCA_029745975.1	Corvallis	River	*mdsA, mdsB, qnrB19*	1541	6°51′47.1″ S 35°21′32.1″ W	Col(pHAD28)
GCA_029746015.1	Corvallis	River	*mdsA, mdsB, qnrB19*	1541	6°51′47.1″ S 35°21′32.1″ W	Col(pHAD28)
GCA_029744825.1	Cerro	River	*mdsA, mdsB, qnrB19*	2407	6°51′47.1″ S 35°21′32.1″ W	Col(pHAD28)
GCA_029745075.1	Corvallis	River	*mdsA, mdsB, qnrB19*	1541	6°51′47.1″ S 35°21′32.1″ W	Col(pHAD28)
GCA_029746195.1	Corvallis	Dam water	*mdsA, mdsB, qnrB19*	1541	7°01′34.9″ S 37°56′36.7″ W	Col(pHAD28)
GCA_029746295.1	Corvallis	Dam water	*mdsA, mdsB, qnrB19*	1541	7°01′34.9″ S 37°56′36.7″ W	Col(pHAD28)
GCA_029746515.1	Hadar	Dam water	*aph(3″)‐Ib, aph(6)‐Id, mdsA, mdsB, qnrB19, tet(A)*	33	7°01′34.9″ S 37°56′36.7″ W	Col(pHAD28)
GCA_029746695.1	Anatum	Dam water	*mdsA, mdsB, qnrB19*	64	7°25′56.5″ S 35°33′41.7″ W	Col156, Col(pHAD28)
GCA_029744115.1	Agona	River	*fosA7.2, mdsA, mdsB, qnrB19*	13	6°51′27.8″ S 35°22′02.5″ W	Col(pHAD28)
GCA_031380575.1	Muenchen	Dam water	*mdsA, mdsB, qnrB19*	112	7°25′57.0″ S 35°33′39.2″ W	Col(pHAD28)
GCA_029672705.1	Corvallis	Dam water	*mdsA, mdsB, qnrB19*	1541	6°50′39.7″ S 38°18′56.1″ W	Col440II, ColpVC, Col(pHAD28)
GCA_029672925.1	Schwarzengrund	Dam water	*aph(3″)‐Ib, aph(6)‐Id, mdsA, mdsB, qnrB19, sul2, tet(A)*	96	6°50′45.1″ S 38°18′40.1″ W	Col(pHAD28)
GCA_029673025.1	Muenchen	Dam water	*mdsA, mdsB, qnrB19*	112	6°39′35.5″ S 35°40′34.0″ W	Col(pHAD28)
GCA_029678185.1	Schwarzengrund	Dam water	*mdsA, mdsB, qnrB19*	96	6°39′30.3″ S 35°40′39.4″ W	Col(pHAD28)
GCA_029673045.1	Schwarzengrund	Dam water	*aph(3″)‐Ib, aph(6)‐Id, mdsA, mdsB, qnrB19, sul2, tet(A)*	96	6°50′45.1″ S 38°18′40.1″ W	Col(pHAD28)
GCA_029672985.1	Schwarzengrund	Dam water	*mdsA, mdsB, qnrB19*	96	6°50′45.1″ S 38°18′40.1″ W	Col(pHAD28)
GCA_029673385.1	Hadar	River	*aph(3″)‐Ib, aph(6)‐Id, mdsA, mdsB, qnrB19, tet(A)*	33	6°51′47.1″ S 35°21′32.1″ W	Col(pHAD28)
GCA_029673725.1	Mbandaka	River	*mdsA, mdsB, qnrB19*	413	6°51′27.8″ S 35°22′02.5″ W	Col(pHAD28)
GCA_029668395.1	Corvallis	River	*mdsA, mdsB, qnrB19*	1541	6°51′47.1″ S 35°21′32.1″ W	Col440II, Col(pHAD28)
GCA_029668025.1	Corvallis	River	*mdsA, mdsB, qnrB19*	1541	6°51′47.1″ S 35°21′32.1″ W	Col440II, Col(pHAD28)
GCA_029673765.1	Corvallis	River	*mdsA, mdsB, qnrB19*	1541	6°51′47.1″ S 35°21′32.1″ W	Col440II, IncFIB(pHCM2), Col(pHAD28)
GCA_029668165.1	Corvallis	River	*mdsA, mdsB, qnrB19*	1541	7°01′00.3″ S 37°59′07.8″ W	Col(pHAD28)
GCA_029674005.1	Corvallis	River	*mdsA, mdsB, qnrB19*	1541	7°01′00.3″ S 37°59′07.8″ W	Col(pHAD28)
GCA_029674065.1	Muenchen	River	*mdsA, mdsB, qnrB19*	112	7°01′00.3″ S 37°59′07.8″ W	Col(pHAD28)
GCA_029674085.1	Molade or Wippra	River	*fosA7.4, mdsA, mdsB, qnrB19*	544	7°01′00.3″ S 37°59′07.8″ W	Col(pHAD28)
GCA_029668635.1	Ohio	River	*mdsA, mdsB, qnrB19*	329	7°01′00.3″ S 37°59′07.8″ W	Col(pHAD28)
GCA_029668905.1	Kiambu	River	*mdsA, mdsB, qnrB19*	309	7°01′00.3″ S 37°59′07.8″ W	Col(pHAD28)
GCA_025537675.1	Hadar	River	*aph(3″)‐Ib, aph(6)‐Id, mdsA, mdsB, qnrB19, tet(A)*	33	6°51′47.1″ S 35°21′32.1″ W	Col(pHAD28)
GCA_025537775.1	Hadar	River	*aph(3″)‐Ib, aph(6)‐Id, mdsA, mdsB, qnrB19, tet(A)*	33	6°51′47.1″ S 35°21′32.1″ W	Col(pHAD28)
GCA_025540375.1	Hadar	Dam water	*aph(3″)‐Ib, aph(6)‐Id, mdsA, mdsB, qnrB19, tet(A)*	33	6°55′03.9″ S 35°40′32.2″ W	Col(pHAD28)
GCA_025537735.1	Hadar	Dam water	*aph(3″)‐Ib, aph(6)‐Id, mdsA, mdsB, qnrB19, tet(A)*	33	6°55′03.9″ S 35°40′32.2″ W	Col(pHAD28)
GCA_025540395.1	Hadar	Dam water	*aph(3″)‐Ib, aph(6)‐Id, mdsA, mdsB, qnrB19, tet(A)*	33	6°55′03.9″ S 35°40′32.2″ W	Col(pHAD28)
GCA_025512155.1	Muenchen	Dam water	*mdsA, mdsB, qnrB19*	112	6°51′22.5″ S 38°21′05.5″ W	Col(pHAD28)
GCA_025512135.1	Corvallis	Dam water	*mdsA, mdsB, qnrB19*	1541	6°51′22.5″ S 38°21′05.5″ W	Col(pHAD28)
GCA_025510715.1	Muenchen	Dam water	*mdsA, mdsB, qnrB19*	112	6°51′22.5″ S 38°21′05.5″ W	Col(pHAD28)
GCA_025401835.1	Hadar	Dam water	*aph(3″)‐Ib, aph(6)‐Id, mdsA, mdsB, qnrB19, tet(A)*	33	7°31′15.9″ S 36°09′57.7″ W	Col(pHAD28)
GCA_029672705.1	Corvallis	Dam water	*mdsA, mdsB, qnrB19*	1541	6°50′39.7″ S 38°18′56.1″ W	Col440II, ColpVC, Col(pHAD28)
GCA_025401735.1	Hadar	Dam water	*aph(3″)‐Ib, aph(6)‐Id, mdsA, mdsB, qnrB19, tet(A)*	33	7°31′15.9″ S 36°09′57.7″ W	Col(pHAD28)
GCA_025401975.1	Hadar	Dam water	*aph(3″)‐Ib, aph(6)‐Id, mdsA, mdsB, qnrB19, tet(A)*	33	7°31′15.9″ S 36°09′57.7″ W	Col(pHAD28)
GCA_025402175.1	Hadar	Dam water	*aph(3″)‐Ib, aph(6)‐Id, mdsA, mdsB, qnrB19, tet(A)*	33	7°31′15.9″ S 36°09′57.7″ W	Col(pHAD28)
GCA_025402295.1	Corvallis	River	*mdsA, mdsB, qnrB19*	1541	6°51′47.1″ S 35°21′32.1″ W	Col440II, Col(pHAD28)
GCA_025402355.1	Hadar	Dam water	*aph(3″)‐Ib, aph(6)‐Id, mdsA, mdsB, qnrB19, tet(A)*	33	7°31′15.9″ S 36°09′57.7″ W	Col(pHAD28)
GCA_025402315.1	Hadar	Dam water	*aph(3″)‐Ib, aph(6)‐Id, mdsA, mdsB, qnrB19, tet(A)*	33	7°31′15.9″ S 36°09′57.7″ W	Col(pHAD28)
GCA_025402395.1	Corvallis	River	*mdsA, mdsB, qnrB19*	1541	6°51′47.1″ S 35°21′32.1″ W	Col440II, Col(pHAD28)
GCA_025402515.1	Hadar	Dam water	*aph(3″)‐Ib, aph(6)‐Id, mdsA, mdsB, qnrB19, tet(A)*	33	7°31′15.9″ S 36°09′57.7″ W	Col(pHAD28)
GCA_025402475.1	Hadar	Dam water	*aph(3″)‐Ib, aph(6)‐Id, mdsA, mdsB, qnrB19, tet(A)*	33	7°31′15.9″ S 36°09′57.7″ W	Col(pHAD28)
GCA_025402455.1	Hadar	Dam water	*aph(3″)‐Ib, aph(6)‐Id, mdsA, mdsB, qnrB19, tet(A)*	33	7°31′15.9″ S 36°09′57.7″ W	Col(pHAD28)
GCA_025402415.1	Hadar	Dam water	*aph(3″)‐Ib, aph(6)‐Id, mdsA, mdsB, qnrB19, tet(A)*	33	7°31′15.9″ S 36°09′57.7″ W	Col(pHAD28)
GCA_025402635.1	Hadar	Dam water	*aph(3″)‐Ib, aph(6)‐Id, mdsA, mdsB, qnrB19, tet(A)*	33	7°31′15.9″ S 36°09′57.7″ W	Col(pHAD28)
GCA_025402695.1	Hadar	Dam water	*aph(3″)‐Ib, aph(6)‐Id, mdsA, mdsB, qnrB19, tet(A)*	33	7°31′15.9″ S 36°09′57.7″ W	Col(pHAD28)
GCA_023802615.1	Muenchen	Dam water	*mdsA, mdsB, qnrB19*	112	7°04′34.2″ S 37°58′43.3″ W	Col(pHAD28)
GCA_023801055.1	Corvallis	Dam water	*mdsA, mdsB, qnrB19*	1541	6°55′45.2″ S 35°40′43.6″ W	Col(pHAD28)
GCA_023731255.1	Corvallis	Dam water	*mdsA, mdsB, qnrB19*	1541	6°55′03.9″ S 35°40′32.2″ W	Col440II, Col(pHAD28)
GCA_023731275.1	Muenchen	Dam water	*mdsA, mdsB, qnrB19*	112	7°01′36.3″ S 37°56′34.4″ W	Col(pHAD28)
GCA_023801535.1	Corvallis	Dam water	*mdsA, mdsB, qnrB19*	1541	6°55′45.2″ S 35°40′43.6″ W	Col(pHAD28)
GCA_024739785.1	Corvallis	Dam water	*mdsA, mdsB, qnrB19*	1541	6°55′45.2″ S 35°40′43.6″ W	Col(pHAD28)
GCA_023733765.1	Corvallis	River	*mdsA, mdsB, qnrB19*	1541	6°51′47.1″ S 35°21′32.1″ W	Col(pHAD28)
GCA_023733215.1	Corvallis	Dam water	*mdsA, mdsB, qnrB19*	1541	6°55′45.2″ S 35°40′43.6″ W	Col(pHAD28)
GCA_023801315.1	Corvallis	Dam water	*mdsA, mdsB, qnrB19*	1541	6°55′45.2″ S 35°40′43.6″ W	Col(pHAD28)
GCA_023799915.1	Corvallis	River	*mdsA, mdsB, qnrB19*	1541	6°51′47.1″ S 35°21′32.1″ W	Col(pHAD28)
GCA_023733125.1	Corvallis	River	*mdsA, mdsB, qnrB19*	1541	6°51′47.1″ S 35°21′32.1″ W	Col(pHAD28)
GCA_023731135.1	Muenchen	Dam water	*mdsA, mdsB, qnrB19*	112	7°01′36.3″ S 37°56′34.4″ W	Col(pHAD28)
GCA_023799955.1	Corvallis	Dam water	*mdsA, mdsB, qnrB19*	1541	6°55′45.2″ S 35°40′43.6″ W	Col(pHAD28)
GCA_023493845.1	Hadar	River	*aph(3″)‐Ib, aph(6)‐Id, mdsA, mdsB, qnrB19, tet(A)*	33	6°51′47.1″ S 35°21′32.1″ W	Col(pHAD28)
GCA_023493825.1	Hadar	River	*aph(3″)‐Ib, aph(6)‐Id, mdsA, mdsB, qnrB19, tet(A)*	33	6°51′47.1″ S 35°21′32.1″ W	Col(pHAD28)
GCA_023494505.1	Hadar	River	*aph(3″)‐Ib, aph(6)‐Id, mdsA, mdsB, qnrB19, tet(A)*	33	6°51′47.1″ S 35°21′32.1″ W	Col(pHAD28)
GCA_023494105.1	Braenderup	River	*mdsA, mdsB, qnrB19*	22	6°51′27.8″ S 35°22′02.5″ W	Col(pHAD28)
GCA_023494565.1	Hadar	River	*aph(3″)‐Ib, aph(6)‐Id, mdsA, mdsB, qnrB19, tet(A)*	33	6°51′47.1″ S 35°21′32.1″ W	Col(pHAD28)
GCA_023494225.1	Hadar	River	*aph(3″)‐Ib, aph(6)‐Id, mdsA, mdsB, qnrB19, tet(A)*	33	6°51′47.1″ S 35°21′32.1″ W	Col(pHAD28)
GCA_023494325.1	Hadar	River	*aph(3″)‐Ib, aph(6)‐Id, mdsA, mdsB, qnrB19, tet(A)*	33	6°51′47.1″ S 35°21′32.1″ W	Col(pHAD28)
GCA_023494265.1	Braenderup	Dam water	*mdsA, mdsB, qnrB19*	22	6°51′47.1″ S 35°21′32.1″ W	Col(pHAD28)
GCA_023494585.1	Braenderup	Dam water	*mdsA, mdsB, qnrB19*	22	6°51′47.1″ S 35°21′32.1″ W	Col(pHAD28)
GCA_023494545.1	Hadar	River	*aph(3″)‐Ib, aph(6)‐Id, mdsA, mdsB, qnrB19, tet(A)*	33	6°51′47.1″ S 35°21′32.1″ W	Col(pHAD28)
GCA_023494725.1	Braenderup	River	*mdsA, mdsB, qnrB19*	22	6°51′47.1″ S 35°21′32.1″ W	Col(pHAD28)
GCA_023496105.1	Muenchen	Dam water	*aac(3)‐*Via, *aadA1, aph(3′)‐Ia, fosL1, mdsA, mdsB, qnrB19, sul1, tet(A)*	112	7°01′36.3″ S 37°56′34.4″ W	IncHI2, IncHI2A
GCA_023496205.1	Braenderup	River	*mdsA, mdsB, qnrB19*	22	6°51′47.1″ S 35°21′32.1″ W	Col(pHAD28)
GCA_023730895.1	Hadar	Dam water	*aph(3″)‐Ib, aph(6)‐Id, mdsA, mdsB, qnrB19, tet(A)*	33	6°51′47.1″ S 35°21′32.1″ W	Col(pHAD28)
GCA_023496265.1	Braenderup	River	*mdsA, mdsB, qnrB19*	22	6°51′47.1″ S 35°21′32.1″ W	Col(pHAD28)
GCA_023496425.1	Corvallis	River	*mdsA, mdsB, qnrB19*	1541	6°51′47.1″ S 35°21′32.1″ W	Col440II, IncFIB(pHCM2)
GCA_023496585.1	Muenchen	Dam water	*aac(3)‐*Via, *aadA1, aph(3′)‐Ia, fosL1, mdsA, mdsB, qnrB19, sul1, tet(A)*	112	7°01′36.3″ S 37°56′34.4″ W	IncHI2, IncHI2A
GCA_023496725.1	Braenderup	Dam water	*mdsA, mdsB, qnrB19*	22	6°51′16.4″ S 35°18′03.9″ W	Col(pHAD28)
GCA_023498525.1	Braenderup	River	*mdsA, mdsB, qnrB19*	22	6°51′47.1″ S 35°21′32.1″ W	Col(pHAD28)
GCA_023497145.1	Muenchen	Dam water	*aac(3)‐*Via, *aadA1, aph(3′)‐Ia, fosL1, mdsA, mdsB, qnrB19, sul1, tet(A)*	112	7°01′36.3″ S 37°56′34.4″ W	IncHI2, IncHI2A
GCA_023732545.1	Muenchen	Dam water	*aadA2, bla*_*TEM‐1*_, *floR, mdsA, mdsB, qnrS1, tet(A), lnu(F)*	112	6°55′03.9″ S 35°40′32.2″ W	IncR
GCA_023734055.1	Muenchen	Dam water	*aadA2, bla*_*TEM‐1*_, *floR, mdsA, mdsB, qnrS1, tet(A), lnu(F)*	112	6°55′03.9″ S 35°40′32.2″ W	IncR
GCA_023733065.1	Muenchen	Dam water	*aadA2, bla*_*TEM‐1*_, *floR, mdsA, mdsB, qnrS1, tet(A), lnu(F)*	112	6°55′03.9″ S 35°40′32.2″ W	IncR
GCA_023837455.1	Muenchen	Dam water	*aadA2, bla*_*TEM‐1*_, *floR, mdsA, mdsB, qnrS1, tet(A), lnu(F)*	112	6°55′03.9″ S 35°40′32.2″ W	IncR

### 2.4 Genomic Sequencing and Data Analysis

*S. enterica* isolates were whole‐genome sequenced on a NextSeq Illumina platform at the Center for Food Safety and Applied Nutrition (CFSAN, now Human Foods Program), FDA. DNA extraction was performed using a commercial kit (DNA Blood and Tissue kit, Qiagen, Germantown) according to the manufacturer's guidelines. Genomic DNA of the isolates was sequenced at a 300‐bp paired‐end‐read using the Illumina DNA Prep library preparation kit at the NextSeq platform (Illumina, San Diego, CA). FastQ files were uploaded into CLC Genomics Workbench (CLC Bio, Qiagen, Aarhus, Denmark) to check the quality of the sequences and ensure the non‐contamination of the reads. Afterwards, *de novo* assembly was performed using the same software. All sequences have been deposited at the NCBI, and their accession numbers are listed in Table 1.

The assemblies were analysed for plasmidome and multilocus sequence typing using default settings of PlasmidFinder 2.1 and MLST 2.0 databases, respectively, available at the Center for Genome Epidemiology (http://www.genomicepidemiology.org/). Furthermore, we used NCBI's AMRFinderPlus to identify antimicrobial resistance genes (ARGs). Antimicrobial resistance genes (ARGs) were identified using AMRFinderPlus v3.10 (https://github.com/ncbi/amr), with default settings. Detection thresholds included a minimum of 90% sequence identity and 50% gene coverage, ensuring both specificity and sensitivity in gene identification. Lastly, all isolates were serotyped *in silico* using default settings in SeqSero 1.2 (http://www.genomicepidemiology.org/).

### 2.5 Phylogenetic Analysis

The genetic relatedness between PMQR‐harbouring *S. enterica* isolates was investigated by means of core genome multilocus sequence typing (cgMLST) using cgMLSTFinder 1.1 (https://cge.cbs.dtu.dk/services/cgMLSTFinder/). The parameters used and tree visualisation were carried out as per Alikhan et al. (2018). cgMLST was chosen over SNP‐based methods due to its ability to provide high‐resolution, standardised genome typing across diverse *S. enterica* serovars, allowing robust comparison of genetic relatedness while minimising alignment artefacts and computational demands, and making it well‐suited for environmental surveillance studies involving large datasets (Alikhan et al. 2018).

## 3 Results

*S. enterica* was recovered from 175 of the 230 (76.08%) water samples, resulting in 2903 isolates, from which 938 were submitted to whole genome sequencing. After genome assembly and further downstream analyses, 130 (13.9%) isolates were confirmed to harbour PMQR determinants. Among these, 124 (95.4%) harboured *qnrB19*, while six (4.6%) harboured *qnrS1*.

The *qnrB19*‐harbouring isolates belonged to 14 different *S. enterica* serovars, with the largest representation from *S.* Corvallis (*n* = 28), *S*. Hadar (*n* = 27), *S*. Muenchen (*n* = 25) and *S*. Schwarzengrund (*n* = 18). Additional serovars included *S*. Braenderup (*n* = 8), *S*. Javiana (*n* = 7), *S*. Agona (*n* = 5), *S*. Anatum (*n* = 4), *S*. Cerro (*n* = 3), *S*. Minnesota (*n* = 1), *S*. Mbandaka (*n* = 1), *S*. Molade or Wippra (*n* = 1), *S*. Ohio (*n* = 1) and *S*. Kiambu (*n* = 1). On the other hand, *qnrS1* was identified in six non‐duplicate *S*. Muenchen isolates (Table 1).

PMQR determinants were detected across all three hydrographic basins investigated. The *qnrB19* gene was reported in 45 isolates (36.3%) from the Piranhas river basin, 41 (33.0%) from the Paraíba basin and 38 (30.7%) from the Mamanguape basin. *S.* Corvallis and *S*. Hadar harbouring *qnrB19* were predominantly recovered from dams adjacent to intensive poultry and livestock farms within the Piranhas basin. Conversely, *qnrS1*‐positive isolates demonstrated a more restricted geographical distribution, confined to two sampling sites within the Paraíba river basin.

In addition, several antimicrobial resistance genes were identified in PMQR‐harbouring isolates, conferring resistance to a wide range of antimicrobials, including aminoglycosides [*aac(3)‐*Via, *aadA1*, *aph(3″)‐Ib*, *aph(6)‐Id*, *aph(3′)‐Ia*], β‐lactams [*bla*_CMY‐2_], multidrug efflux pump [*mdsA*, *mdsB*], sulphonamide [*sul1*, *sul2*], fosfomycin [*fosA7.2*, *fosL1*] and tetracycline [*tet(A)*] (Table 1).

**FIGURE 2:**
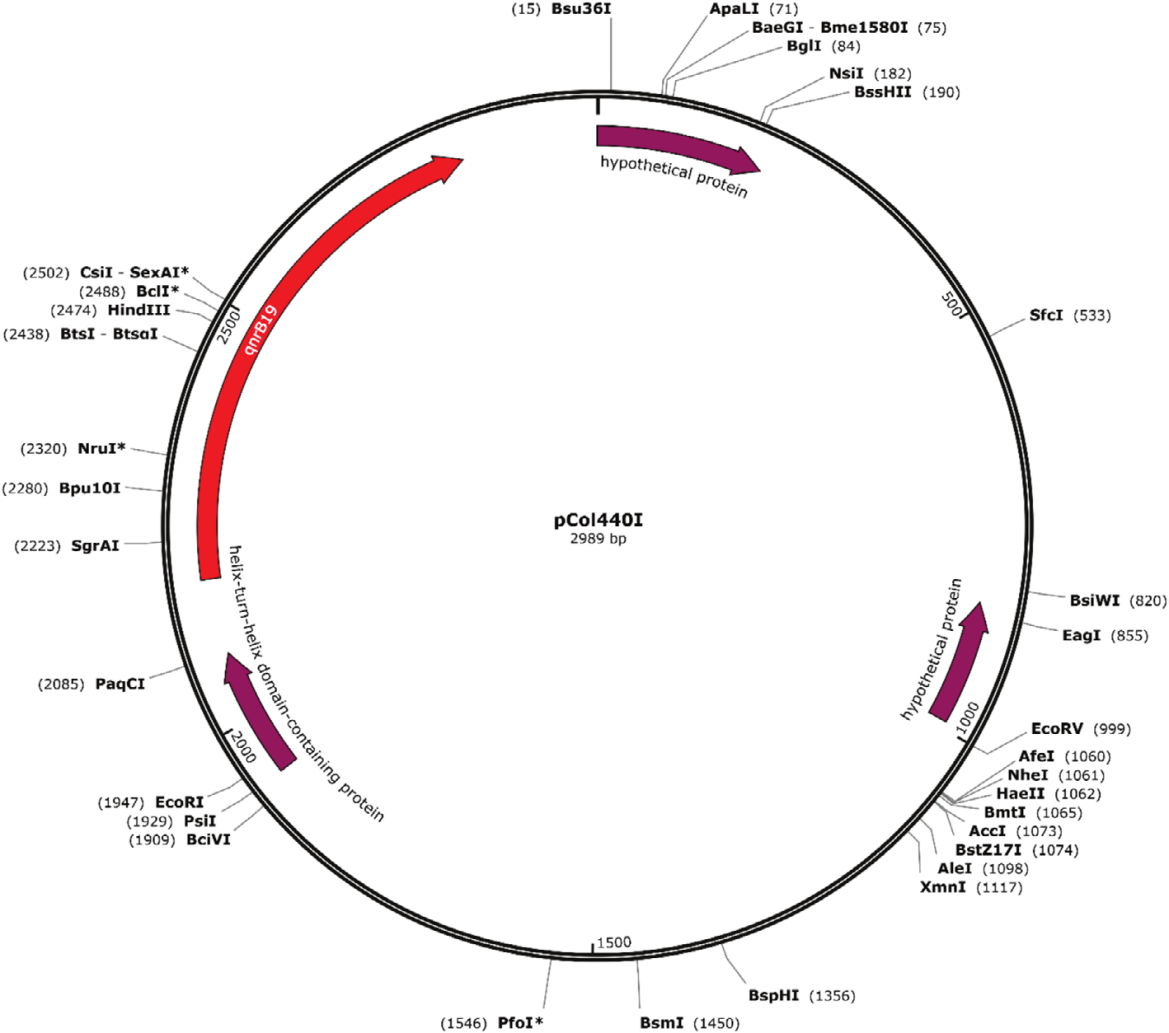
Plasmid harbouring *qnrB19* in *Salmonella enterica* serovars.

**FIGURE 3:**
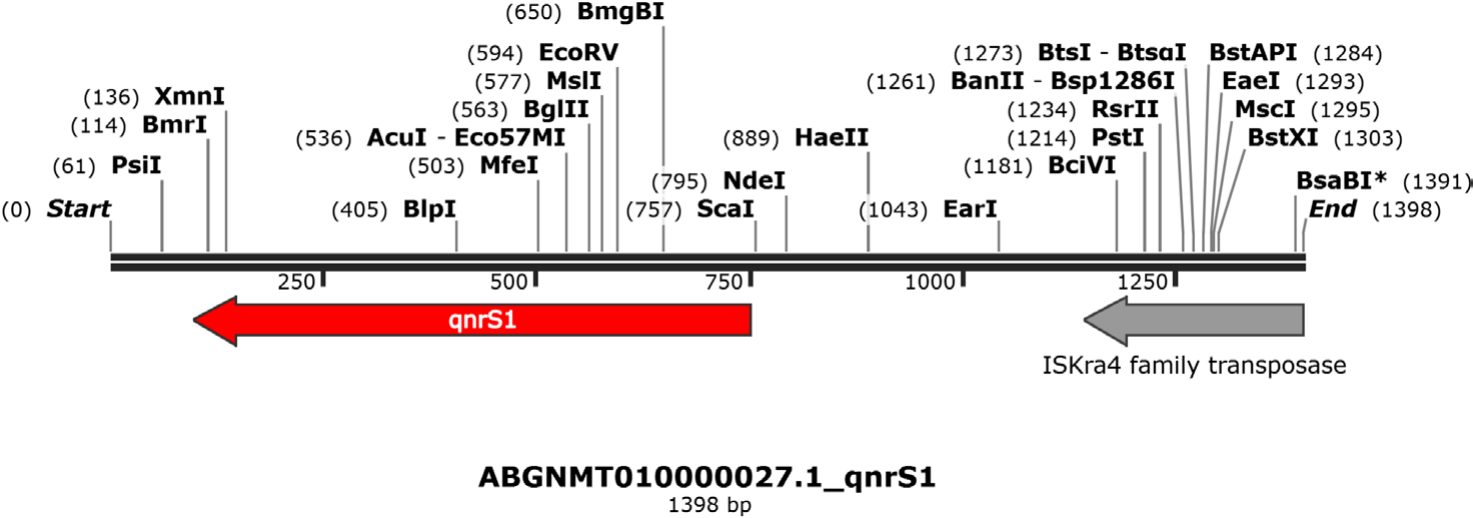
Genetic context of *qnrS1* in *Salmonella enterica* serovars.

The genetic context characterisation of the PMQR determinants revealed that the *qnrB19* gene was located on a 2989 bp plasmid (Figure 2), while the *qnrS1* gene was surrounded by an ISkra4 element measuring 1398 bp (Figure 3). *In silico* analyses revealed the presence of several plasmids, including Col(pHAD28), Col156, IncC, ColpVC, Col440II, IncFIB(pHCM2), IncHI2, IncHI2A and IncR (Table 1).

**FIGURE 4:**
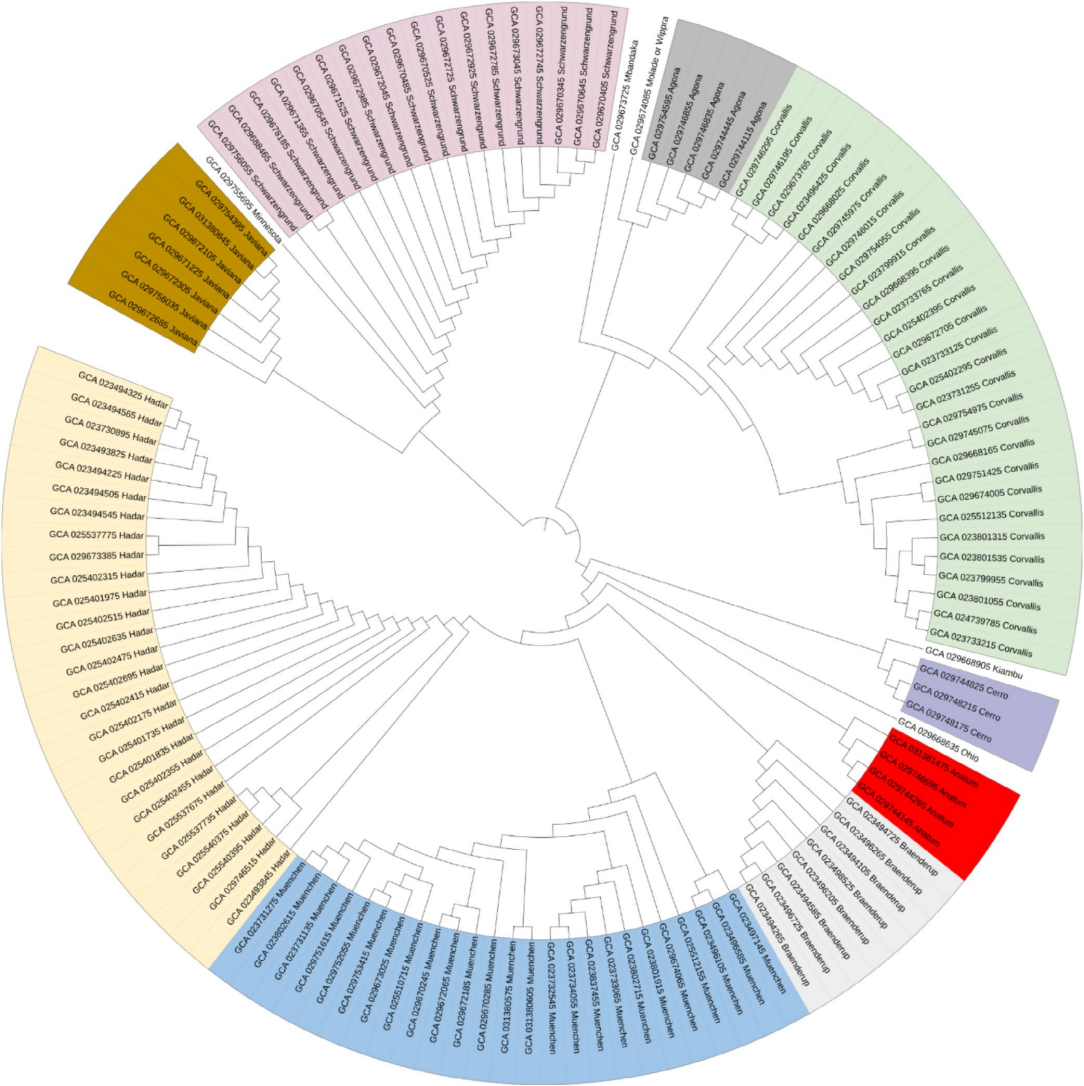
Phylogenetic tree based on core genome multilocus sequence typing (cgMLST) of 130 *Salmonella enterica* isolates from surface waters in Paraíba, Brazil. The phylogeny is rooted at the midpoint.

Multi‐locus sequencing typing (MLST) revealed that each serovar was assigned to a specific ST, such as *S*. Anatum ST64, *S*. Agona ST13, *S*. Schwarzengrund ST96, *S*. Muenchen ST112, *S*. Javiana ST1674, *S*. Minnesota ST548, *S.* Corvallis ST1541, *S*. Cerro ST367, *S*. Hadar ST33, *S*. Mbandaka ST413, *S*. Molade or Wippra ST544, *S*. Ohio ST329, *S*. Kiambu ST309 and *S*. Braenderup ST22 (Table 1). Phylogenetic analysis showed each serovar was represented by a monophyletic clade on the reconstructed cgMLST (Figure 4).

## 4 Discussion

We uncovered a high prevalence of PMQR determinants among *S. enterica* isolated from surface water samples from the main hydrographic basins in Paraíba, Northeastern Brazil. These epidemiological observations suggest significant spatial variation in serovar and resistance determinant distributions across the investigated geographical area, potentially attributable to localised agricultural practices, agricultural runoffs and precipitation‐induced watershed contamination events.

Similar findings have been reported in various geographic contexts, reinforcing the potential role of aquatic environments as reservoirs and dissemination pathways of PMQR‐harbouring *S. enterica* . In Thailand, a study analysing 333 canal water samples revealed that 35.3% of *Salmonella* isolates carried PMQR genes, with *qnr*S being the most frequently detected variant (Toyting et al. 2024). In Latin America, *qnrB19* was identified as one of the most prevalent resistance determinants in *S*. Braenderup and *S*. Muenchen recovered from surface waters in Brazil and Mexico (Chen et al. 2024). Interestingly, the genes *qnrS1* and *qnrB19* have been reported in *E. coli* from seawater and seagull faeces collected on Berlenga Island (Alves et al. 2014).

The frequent detection of *qnrB19* and *qnrS1* in a diversity of *S. enterica* recovered from surface waters confirms the global emergence and promiscuity of PMQR genes across diverse serovars and environments. Moreover, it highlights the putative role of environmental water in the dissemination of antimicrobial resistance through contamination cycles amplified by anthropogenic activities in both rural and urban environments. Indeed, *qnr*B19‐harbouring *S. enterica* strains have been frequently reported in livestock and animal‐derived foodstuffs globally. For instance, *S*. Muenster harbouring *qnrB19* on a Col440I plasmid has been reported in an abattoir in Nigeria (Aworh et al. 2024). In Brazil, there have been reports of *S*. Newport co‐harbouring *bla*_CMY‐2_, *qnrB19* and *mcr‐9*, isolated from diarrhoeic faeces of a foal (Braga et al. 2023). Additionally, *qnrB19* has also been detected in the rare *S. enterica* serovar Isangi, demonstrating the widespread promiscuity of this gene across diverse serovars (Monte et al. 2021; Vilela et al. 2023). Other investigations in Brazil reported *qnrB19*‐harbouring strains in broiler chickens associated with different serovars, such as *S*. Minnesota, *S. infantis* , *S*. Schwarzengrund, *S*. Newport, *S*. Brandenburg and *S*. Heidelberg. The presence of *qnrS* has been documented in food‐related *S. typhimurium* isolates and human‐derived isolates in China (Yang et al. 2023), suggesting a potential link between food contamination and the dissemination of resistance genes.

The widespread occurrence of PMQR in *S. enterica* of different serovars isolated from human clinical samples, wild and domestic animals and the environment, including foodstuffs (Monte et al. 2019; Moreno‐Switt et al. 2019; Al‐Gallas et al. 2021; Vilela et al. 2023), supports the view that surface waters, particularly those impacted by agricultural runoff, may serve as significant reservoirs for resistant pathogens. This is particularly concerning because these waters can potentially lead to widespread contamination of agricultural irrigation systems, recreational waters and drinking water sources, amplifying the risk of human exposure to antibiotic‐resistant pathogens.

The presence of other determinants conferring resistance to clinically important antimicrobials, such as aminoglycosides [*aac(3)‐*Via, *aadA1*, *aph(3″)‐Ib*, *aph(6)‐Id*, *aph(3′)‐Ia*], β‐lactams [*bla*_CMY‐2_] multidrug efflux pump [*mdsA*, *mdsB*], sulphonamide [*sul1*, *sul2*] fosfomycin [*fosA7.2*, *fosL1*] and tetracycline [*tet(A)*] reveals a broad spectrum of resistance genes, highlighting the multidrug resistance potential of the environmental *S. enterica* , posing a significant public health risk. Notably, high prevalence of resistance to fosfomycin has been reported in *S. enterica* serovars (Monte et al. 2023).

The identification of several plasmids, including IncC, Col(pHAD28) and IncFIB(pHCM2), highlights the significant role these mobile genetic elements play in facilitating horizontal gene transfer (HGT). Plasmids of the IncC incompatibility group are particularly noteworthy, as they are known to harbour multidrug resistance (MDR) genes and have been widely associated with the global spread of *bla*_CMY‐2_ and other β‐lactamase genes. These plasmids are highly mobile and capable of transferring genes across bacterial species and even genera, contributing to the rapid dissemination of resistance traits in diverse environments (Carattoli 2003). Similarly, Col(pHAD28) plasmids, which have been identified in *E. coli* and *Salmonella* isolates, are frequently associated with *qnr* genes, such as *qnrB19*, conferring fluoroquinolone resistance. These plasmids are distributed globally, including in agricultural settings, and have been implicated in the spread of PMQR genes from environmental reservoirs to clinical pathogens.

The presence of the *qnrB19* gene on a 2989 bp plasmid is consistent with previous findings (Monte et al. 2019). This small plasmid was identical to the *qnrB19* plasmid identified in *E. coli* in Brazil in 2016 (KX452393.1). It also shared 99% identity with *qnrB19* plasmids found in *S*. Muenchen in the United States in 2017 (KY991368.1) and in *S. enterica* serovars in Canada in 2018 (CP030230.1). This highlights the remarkable conservation and potential for global dissemination of this plasmid. The plasmid's minimal size and high nucleotide sequence conservation across diverse geographic locations underscore its enhanced mobility potential and capacity for global dissemination, thereby facilitating the spread of fluoroquinolone resistance determinants.

The *qnrB19* detection in plasmids harboured by phylogenetically diverse bacterial species emphasises the remarkable genetic adaptability and horizontal transferability of this resistance determinant across heterogeneous environmental niches. This observation aligns with previous molecular epidemiological investigations in Brazil (Monte et al. 2019; Vilela et al. 2023), highlighting the transnational nature of PMQR dissemination. The plasmid‐mediated transmission of resistance genes between clinical, animal and environmental strains appears to be a common mechanism in diverse geographical settings, reinforcing the need for global surveillance to monitor and control the spread of fluoroquinolone resistance. Furthermore, small *qnrB19*‐carrying plasmids can be transferred between different *S. enterica* serotypes through a P22‐mediated transduction (Moreno‐Switt et al. 2019).

**FIGURE 5:**
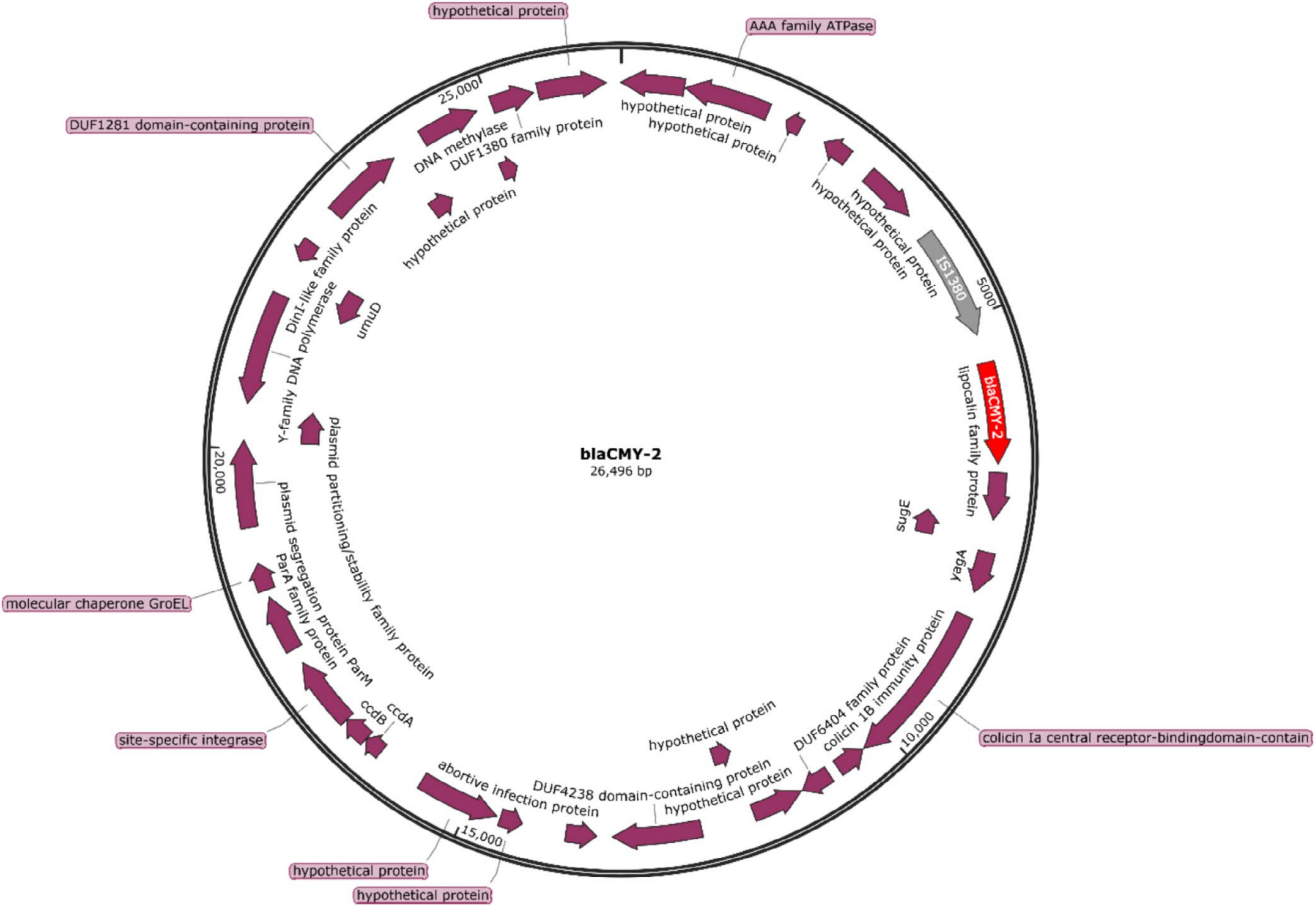
Plasmid harbouring *bla*_CMY‐2_ in *S*. Minnesota.

In contrast, the 1398 bp‐ISkra4 element surrounding the *qnrS1* gene differs from a previous report (Monte et al. 2019) showing that the *qnrS1* gene was flanked by different insertion sequences. The presence of ISkra4 suggests potential transposition and mobilisation of the *qnrS1* gene, which could enhance its spread across bacterial populations and emphasise the genetic diversity of PMQR determinants in *Salmonella*. The mobilisation of these genes via mobile genetic elements like ISs can contribute to the horizontal gene transfer of resistance genes between environmental and clinical bacterial strains. In addition, one *S*. Minnesota presented a closed plasmid with 26,496 bp in size harbouring *bla*_CMY‐2_, as shown in Figure 5.

Among the ARGs identified, *bla*_CMY‐2_ is of particular concern due to its role in conferring resistance to third‐generation cephalosporins, considered critically important antibiotics (WHO 2024). The presence of this gene in a closed plasmid within an *S*. Minnesota isolate underscores the potential for stable horizontal gene transfer of extended‐spectrum β‐lactamase (ESBL) resistance in environmental reservoirs. These drugs are often used as first‐line therapies for salmonellosis in both human and veterinary medicine. The identification of these genes in environmental *S. enterica* , especially in surface waters, suggests the potential role of agricultural runoffs in the dissemination and maintenance of environmental reservoirs of antimicrobial resistant genes to critically important drugs.

Additional clinically relevant ARGs identified include *fosA7.2* and *fosL1*, associated with fosfomycin resistance, and *aac(3)‐*Via and *aph(3′)‐Ia*, conferring resistance to aminoglycosides. These multi‐drug resistance profiles highlight the complexity and adaptability of environmental *S. enterica* populations and stress the importance of integrating environmental AMR surveillance into One Health frameworks.

Clustering was primarily driven by serovar and sequence types, with no significant correlation to resistance profile, year of isolation, source or geographic location. These findings corroborate previous studies (Monte et al. 2019, 2021), suggesting a degree of clonality within certain serovars. The phylogenetic analysis using cgMLST revealed that isolates clustered into monophyletic clades corresponding to their respective serovars. This tight clustering suggests clonal expansion of specific lineages within the aquatic environment. Importantly, despite genetic clustering by serovar, there was no apparent association between phylogeny and the presence of resistance genes, sampling period or geographic location. This indicates that resistance determinants such as *qnrB19* and *bla*_CMY‐2_ are likely disseminated via horizontal gene transfer rather than vertical inheritance, possibly through plasmids or mobile genetic elements. The strong serovar‐specific clustering highlights the importance of considering both clonal spread and mobile element‐mediated gene flow in understanding environmental AMR dynamics.

Although this study provides valuable insights into the prevalence and genetic context of PMQR in *S. enterica* from surface waters in Paraíba, Brazil, several limitations should be acknowledged. First, the geographic scope was restricted to selected dams and rivers within a single Brazilian state, which may limit the generalisability of findings to other regions with different agricultural, climatic or sanitation profiles. Second, as a longitudinal design, we have not accounted for potential factors such as rainfall, seasonal farming activities or sporadic contamination events that could affect or may explain the occurrence of PMQR‐ *S. enterica* across the study area. Third, while *in silico* analyses provided detailed insights into ARGs and plasmid types, the study did not include functional assays such as conjugation or expression studies to validate the transferability and activity of these resistance determinants. Lastly, our investigation focused on *S. enterica* only, overlooking other clinically significant waterborne pathogens that could harbour and disseminate antimicrobial resistance genes (ARGs). For instance, *qnrS1* and *qnrB19* were detected in *E. coli* from seawater and seagull faeces collected in the remote archipelago of Berlenga, Portugal (Alves et al. 2014).

Therefore, future research should aim to expand geographic coverage, incorporate metagenomic or transcriptomic approaches and evaluate environmental drivers such as antibiotic residues, nutrient levels and microbial diversity that may influence ARG persistence and spread. Longitudinal studies incorporating weather and land use data would also provide a more comprehensive understanding of the ecological dynamics underlying AMR dissemination in surface waters.

Given the potential for waterborne transmission of antimicrobial‐resistant pathogens, our findings underscore the need for a more holistic, One Health‐oriented approach to enhance the surveillance of surface water bodies, particularly in areas with significant agricultural activities.

In summary, the high frequency and diversity of PMQR‐harbouring *S. enterica* strains reinforce the importance of surveillance and monitoring of antimicrobial resistance in surface waters. These water bodies can function as reservoirs where resistant bacteria from urban areas and livestock farm runoffs can persist and proliferate. Moreover, these resistance determinants can be transferred to indigenous aquatic microbiota through horizontal transfer mechanisms, creating a complex ecological network where resistance genes circulate between agricultural, environmental and human domains. Furthermore, the watershed dynamics allow resistant *Salmonella* to travel considerable distances, potentially contaminating irrigation systems, recreational waters and drinking water sources. Understanding these environmental pathways is essential for developing comprehensive AMR surveillance and mitigation strategies. Further research is needed to elucidate the mechanisms driving the dissemination of resistance determinants and to develop effective interventions to combat antimicrobial resistance in surface water environments, safeguarding water quality and public health.

## Author Contributions

D.F.M.M. writing – original draft, methodology, formal analysis, investigation, data curation, visualization. A.D.L.R. data curation, investigation, writing – original draft, visualization. M.L.P.L. writing – original draft, investigation, visualization. L.A.L., J.M.C, N.J.S. writing – original draft, investigation. X.H., Z.C., E.W.B., M.W.A., R.L.B. funding acquisition, resources, writing – review and editing, validation. M.T. writing – review and editing, validation, supervision. J.M. funding acquisition, resources, project administration, writing – review and editing, validation, funding acquisition, project administration, supervision. C.J.B.O. resources, supervision, project administration, writing – review and editing, validation, funding acquisition, conceptualization.

## Conflicts of Interest

The authors declare no conflicts of interest.

## Data Availability

The data that support the findings of this study are available in National Library of Medicine at https://www.ncbi.nlm.nih.gov/bioproject/?term=PRJNA560080.
